# Behavioral and anatomical characterization of the bilateral sciatic nerve chronic constriction (bCCI) injury: correlation of anatomic changes and responses to cold stimuli

**DOI:** 10.1186/1744-8069-6-7

**Published:** 2010-01-27

**Authors:** Sukdeb Datta, Koel Chatterjee, Robert H Kline, Ronald G Wiley

**Affiliations:** 1 Department of Anesthesiology, Vanderbilt University, Nashville, TN, USA; 2 Laboratory of Experimental Neurology, Tennessee Valley Healthcare System, Department of Veterans Affairs, Nashville, TN, USA; 3 Department of Neurology and Pharmacology, Vanderbilt University, TN, USA

## Abstract

**Background:**

Unilateral constrictive sciatic nerve injury (uCCI) is a common neuropathic pain model. However, the bilateral constrictive injury (bCCI) model is less well studied, and shows unique characteristics. In the present study, we sought to correlate effects of bCCI on nocifensive responses to cold and mechanical stimuli with selected dorsal horn anatomic markers. bCCI or sham ligation of both rat sciatic nerves were followed up to 90 days of behavioural testing. Additional rats sacrificed at 15, 30 and 90 days were used for anatomic analyses. Behavioural tests included hindpaw withdrawal responses to topical acetone, cold plate testing, an operant thermal preference task and hindpaw withdrawal thresholds to mechanical probing.

**Results:**

All nocifensive responses to cold increased and remained enhanced for >45 days. Mechanical withdrawal thresholds decreased for 25 days only. Densitometric analyses of immunoperoxidase staining in the superficial dorsal horn at L4-5 revealed decreased cholecystokinin (CCK) staining at all times after bCCI, decreased mu opiate receptor (MOR) staining, maximal at 15 days, increased neuropeptide Y (NPY) staining only at days 15 and 30, and increased neurokinin-1 receptor (NK-1R) staining at all time points, maximal at 15 days. Correlation analyses at 45 days post-bCCI, were significant for individual rat nocifensive responses in each cold test and CCK and NK-1R, but not for MOR or NPY.

**Conclusions:**

These results confirm the usefulness of cold testing in bCCI rats, a new approach using CCI to model neuropathic pain, and suggest a potential value of studying the roles of dorsal horn CCK and substance P in chronic neuropathic pain. Compared to human subjects with neuropathic pain, responses to cold stimuli in rats with bCCI may be a useful model of neuropathic pain.

## Background

Partial injury to peripheral nerves of rats has been used to investigate mechanisms of chronic neuropathic pain, perhaps modeling human nerve injury pain syndromes. One of the most commonly used models involves unilateral loose ligation of the sciatic nerve with chromic gut sutures (CCI)[1]. This procedure produces ipsilateral reflex hyper-responsiveness to mechanical stimulation which lasts less than four weeks and variable changes in reflex withdrawal to heat. This model (unilateral CCI tested with heat or mechanical withdrawal) has inconsistently predicted clinically useful new treatments for neuropathic pain. The extent to which CCI actually mimics any particular clinical neuropathic pain condition is uncertain [2,3]. This animal model has provided a conspicuously reproducible ground for testing possible treatment interventions for both spontaneous and stimulus evoked pain. However, there is an obvious discrepancy between animal models of peripheral nerve injury and clinical traumatic neuropathy; i.e. the extremely high incidence of "pain like" behavior and facilitated withdrawal reflexes in animals and the relatively rare painful sequelae of nerve injury in humans. Indeed, the most common sensory complaints in clinical peripheral neuropathies are tingling paresthesia and numbness, rather than pain[2]. In general, behavioural testing of withdrawal responses of a "neuropathic" hind paw in different animal models to a short-lasting punctuate prodding of the skin using von Frey filaments and to heat stimuli have gained popularity in the preclinical scientific pain community. The punctuate von Frey induced hind paw withdrawal in the uCCI model has probably very little relationship to the complex experience of dynamic mechanical allodynia elicited with a light moving stimulus that is so common in some neuropathic pain patients [2]. Additionally, heat allodynia is a rare finding in clinical neuropathic pain states [4,5] and is not a problem in the activities of daily life of for patients with neuropathic pain. Therefore, the inclusion of heat-induced reflex hind paw withdrawal to punctate mechanical or heat stimuli as part of the behavioural testing procedure frequently used in animal models of neuropathy lacks a valid clear rationale in light of clinical observations. Lastly, a particular concern is the fact that changes to heat and mechanical stimuli seen with unilateral CCI (uCCI) are transient, lasting four weeks or less, as opposed to clinically important neuropathic pain problems such as complex regional pain syndrome (CRPS) that last for years, often many years. These striking differences in time course raise some concern that short term studies of uCCI may be focusing on initial phenomena idiosyncratic to the procedure rather than long lasting aspects underlying the most important clinical problems.

A recent provocative report using CCI of both sciatic nerves in each rat (bCCI) has shown long lasting increases in nocifensive responses to cold with no change in responses to heat[6]. This important study showed increased reflex and operant nocifensive responses to cold stimuli lasting at least 100 days suggesting a previously unsuspected relevance of cold responses in chronic CCI, as opposed to studying short term (less than four weeks) effects on reflex mechanical paw withdrawal or heat. These observations are reminiscent of the frequent complaint of cold sensitivity from patients with neuropathic disorders further supporting the potential clinical relevance of long term studies of cold sensitivity in the CCI model. Vierck et al [6], using Long Evans female rats, did not test mechanical sensitivity leaving open the possibility that bCCI also might produce long lasting changes in nocifensive responses to mechanical stimuli rather than the transient changes seen in the uCCI. Lastly, Vierck et al. performed all of their experiments on one strain, female Long Evans rats leaving open another possibility that their findings with bCCI were unique to this one strain, a significant concern given clear evidence that neuropathic pain models show considerable variability across rat strains [7-13].

A number of inflammatory mechanisms have been proposed to explain the short term changes seen in the uCCI model. However, there are no studies that might shed light on any anatomic basis for the observed long term behavioural effects of the bCCI. Several workers [14-16] have noted that significant neurochemical and neuroanatomical changes occur in primary sensory neurons and their central projection territories following neuropathic injury such as partial sciatic nerve transaction or uCCI. However, no such information is currently available for the bCCI model. We hypothesized that neuroanatomical changes will occur in the bCCI model, and these changes may differ from other, established neuropathic pain models. We were also interested in following the neuroanatomical changes over time, so that we could evaluate correlations, if any, between changes in behavior and neuroanatomical changes following bCCI. Considering the complex cascade of neurotransmitters involved in neuropathic injury, we decided to focus on four key markers (Cholecystokinin, Neurokinin -1 Receptor, Mu Opioid Receptor and, Neuropeptide Y) as an initial start into dissecting the complex interactions between behavioral and anatomical changes.

In the case of CCK, it is known to be an endogenous anti-opiate. Xu and colleagues found that unilateral spinal nerve ligation led to an increase in CCK in 30 percent of dorsal root ganglia cells fourteen days after the axotomy [17]. Several groups have studied the effects of peripheral nerve injury on spinal mu-opioid receptor (MOR) expression in the rat spinal cord with mixed results. For example, despite dramatic loss of morphine anti-nociceptive activity after L5-L6 spinal nerve ligation (SNL)[18,19], nerve ligation produced only a small decrease in MOR-1 immunoreactivity ipsilateral to the injury and no corresponding changes in ligand binding [20]. Functionally, the role of Neuropeptide Y (NPY) at the spinal level has been studied for more than 15 years using various pain and nerve injury models combined with intrathecal application of NPY agonists and antagonists, suggesting that this peptide has both pro- and anti-nociceptive actions [21-29]. A mouse with genetically deleted NPY-1R has been shown to have a markedly reduced pain threshold [30]. Doyle and Hunt [31] found that neurokinin-1 (NK-1) cells encode the intensity of noxious cooling of the skin. Considering that our behavioural testing encompassed testing for nociceptive responses to cold stimuli (acetone and cold plate); we evaluated changes in the NK-1 receptor after bCCI.

The present study seeks, in rats with bilateral CCI or sham surgery, to determine: 1- if the remarkable behavioural findings of Vierck et al [6] are seen in a different strain of rats, the widely used Sprague Dawley strain; 2- if the observed responses to cold stimuli hold true for topical acetone, a technically simple test to perform; 3- if several key anatomic markers in the superficial dorsal horn thought relevant to neuropathic pain are altered with a time course consistent with observed behavioural changes, and 4- if the time course of changes in mechanical sensitivity of bCCI rats is similar to uCCI or to cold responses in bCCI rats. The results of the present experiments support and **extend **the original observations of Vierck et al; provide evidence that these results are not idiosyncratic to one strain of rats and reveal a somewhat surprising anatomic correlate of the long term behavioural changes in bCCI.

## Results

### General observations

"Spontaneous behaviours" such as lifting the ipsilateral hind limb, which have been interpreted as evidence of spontaneous pain after unilateral CCI were not seen in the present study. No motor deficit was observed, contrary to the mild motor deficit observed in a previous study [6]. This difference in outcome may be related to minor differences in operative technique. Activity levels in home cages and testing enclosures were not obviously altered as revealed by casual observation in home cages. Rats showed normal weight and appetite and did not spontaneously vocalize in contrast to unilateral CCI models. No autotomy was observed. Rats also did not react aversively or aggressively to handling and no spontaneous guarding was observed. We therefore conclude that the bCCI model does not produce overt evidence of chronic ongoing pain or tonic flexion of the hind limb.

### Cold Plate (0.3°C) results

**Figure 1:**
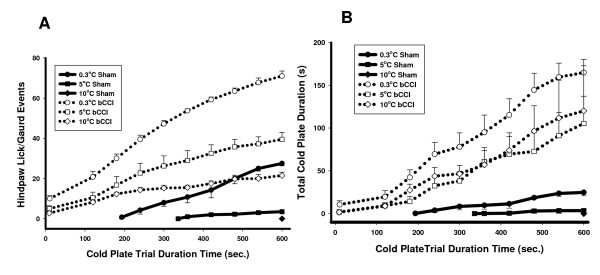
**Group mean behaviors on cold plate 3-4 weeks after bCCI**. Each curve begins at the average first latency and shows the cumulative total number of withdrawal responses. (A) Cumulative hind paw Lick/Guard Events at 0.3, 5 and 10°C. The sham operated rats did not demonstrate any events at 10°C, whereas both 5 and 0.3 C temperatures did elicit some events. On the other hand, bCCI rats showed evidence of increased nocifensive reflex responses at all three plate temperatures (p < 0.001; F = 90.785; df = 1; three-way ANOVA). (B) Total cold plate cumulative nocifensive reflex response duration on three plate temperatures. The sham operated rats did not demonstrate any events at 10°C, whereas both 5 and 0.3 C temperatures did elicit some events. On the other hand, bCCI rats showed increased responding at all three temperatures. Each data point is group mean +/- SEM. (p = 0.023; F = 6.181; df = 1; three-way ANOVA).

In order to better understand reflex response characteristics of cold sensitivity in the bCCI model, we performed a temperature-response curve on the cold plate as shown in Figure 1(A) and 1(B). Control (sham-operated) and bCCI rats were tested at each of three temperatures (0.3°, 5° and 10°C) on the cold plate. Latencies for each hindpaw response (lifting/guarding) and duration of each response, as well as total number of hindpaw lifting/guarding responses were assessed. Total trial duration was 600 seconds. Rats tended to lift (guard) either hindpaw, but licking was very rarely observed.

### Control (sham-operated) rats

As shown in figure 1, control (sham-operated) rats did not respond at all to a plate temperature of 10°C. Mean latency to first response on 5°C was 350 seconds, and on 0.3°C the mean first response latency was 200 seconds.

### bCCI rats

In contrast, there was a very short latency (less than 50 seconds) to the first response in the bCCI group on all three temperatures. The total time spent lifting/guarding (duration) was significantly greater for bCCI rats at all three temperatures (p = 0.023; F = 6.181; df = 1; three-way ANOVA) as were the total number of lifting/guarding responses (p < 0.001; F = 90.785; df = 1; three-way ANOVA).

**Figure 2:**
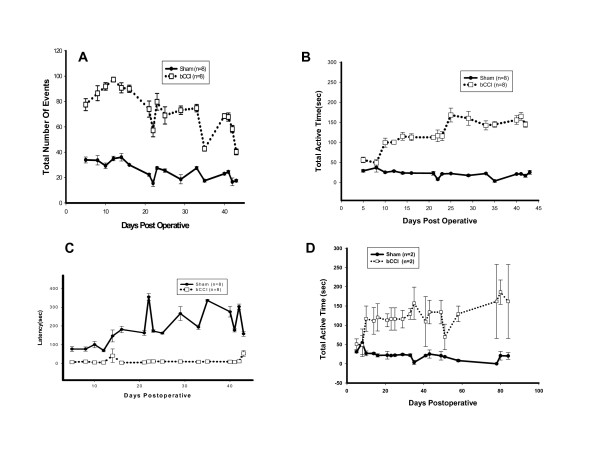
**Time course of changes in cold sensitivity plate responses after bilateral ligation surgery, tested at 0.3°C**. Total number of hindpaw lifts. bCCI rats had increased number of hindpaw lift events compared to sham operated animals. Results were highly significant (p < 0.001; F = 874.58; DF = 1; two-way ANOVA) during the entire testing period. (B) Total active duration of hindpaw lifts. More activity demonstrates presence of increased nocifensive reflex responding to cold. bCCI rats showed a highly significant difference (p < 0.001; F = 510.26; df = 1; two-way ANOVA) from day 9 of testing. (C) First hindpaw lift to cold stimuli. bCCI rats showed a highly significant difference (p < 0.001; F = 970.51; df = 1; two-way ANOVA) throughout the testing period. (D) Total time spent lifting per 600 second trial on a subgroup of 4 rats tested up to 90 day post-bCCI or sham surgery. bCCI rats showed increased time spent lifting compared to shams that persisted for the duration of testing (90 days).

Figure 2 shows the postoperative time course of changes in hindpaw lift/guard responses on the 0.3°C cold plate for up to 45 days after ligation surgery. As mentioned above, each trial lasted 10 minutes (600 seconds). bCCI rats demonstrated increased numbers of responses to cold that persisted throughout the testing period. Cold plate responses peaked at 10-30 days; however results were highly significant throughout the entire testing period (p ≤ 0.001; F = 874.584; df = 1; two- way ANOVA). Similar results were obtained for the total response duration (p ≤ 0.001; F = 510.264; df = 1; two- way ANOVA) and latency to first hindpaw lift/guard (p < 0.001; F = 970.51; df = 1; two- way ANOVA). In the subset of 4 rats who underwent testing for a prolonged period (up to 90 days postoperatively), bCCI rats continued to show increased numbers of hindpaw lifts compared to sham-operated animals.

### Thermal Preference Testing

**Figure 3:**
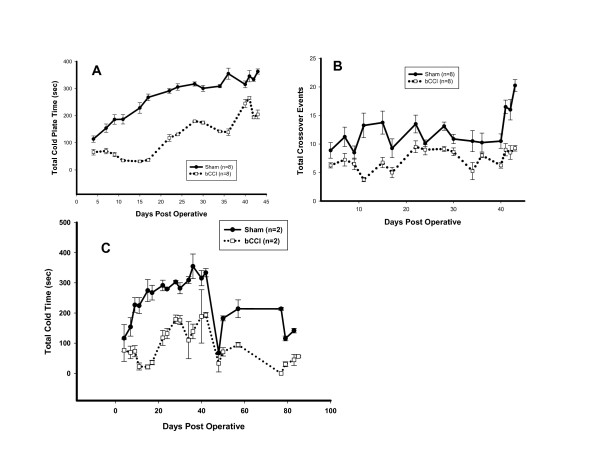
**Operant testing of thermal preference (0.3°C vs. 45°C)**. Rats were allowed to roam freely in a two-chambered shuttle box with different floor temperatures on each side. (A) Total time spent on the cold (0.3°C) side. bCCI rats showed marked preference for the warmer side. Total trial duration was for 10 minutes (600 seconds). (p = 0.004 day 4; p < 0.001; F = 804.83; df = 1; two-way ANOVA day 7 to end of trial period). (B) Total crossover events. Rats were started on either 0.3°C or 45°C in a random fashion on the day of testing to prevent acclimatization. Crossover events were measured to either warm or cold side. bCCI rats showed statistically significant fewer crossover events (p < 0.001; F = 109.9; df = 1; two-way ANOVA) than sham operated rats. Data points are group means +/- SEM. (C) Total time spent on the cold (0.3°C) side in a subgroup of 4 rats tested up to 90 days post-surgery showing bCCI rats had a marked preference for the warmer side, which persisted for the total 90 days. Total trial duration was for 10 minutes (600 seconds).

Figure 3 shows the results of thermal preference testing comparing cold (0.3°C) versus warm (45°C). The primary dependent variable was total time spent on the cold side. Number of crossovers between chambers in either direction also was recorded. Floor temperatures were randomly switched from side to side each day, resulting in being placed on either the hot or the cold side in random fashion to minimize initial response bias. Sham-operated rats showed 50% occupancy on the cold side by day 23. bCCI rats spent less time on the cold side throughout the entire testing period. The bCCI rats never reached 50% occupancy on the cold side during the entire testing period. The difference between bCCI and sham-operated rats was highly significant (p ≤ 0.001; F = 804.83; df = 1; two- way ANOVA) from day 7 and persisted throughout the testing period. As shown in Figure 3B, shams crossed back and forth more frequently (p ≤ 0.001; F = 109.9; df = 1; two- way ANOVA). bCCI rats that were placed on the warm side tended not to cross to the cold side. bCCI rats started on the cold side crossed over to the warm side and tended to stay on the warm side. Similar to results obtained with cold reflex testing, the subset of 4 rats that underwent behavioural testing for up to 90 days continued to show a marked preference for the warm side.

### Acetone Testing

**Figure 4:**
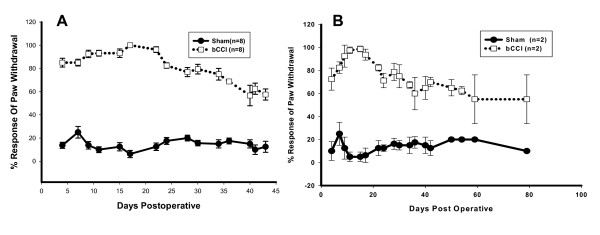
**Enhanced nocifensive responses to acetone application**. (A) Acetone was applied to each separately hindpaw separately (5 trials per side) and results scored as % response. Higher percentage indicates development of cold hyper-reflexia. bCCI rats demonstrated significantly enhanced responsiveness (p < 0.001; F = 2442.5; df = 1; two-way ANOVA) for up to 43 days, which did not return to baseline. Each data point is group mean +/- SEM. (B) Acetone in a subgroup of 4 rats revealed that cold hyperalgesia persisted for up to 80 days.

As shown in Figure 4, rats with bCCI demonstrated increased frequency of responding to hindpaw acetone application compared to controls beginning with post operative day 3 (p < 0.001; F = 2442.5; df = 1; two-way ANOVA). The increased frequency of response to acetone persisted through the end of the study period. Maximal differences between control (sham-operated) and bCCI groups were observed between days 9 and 22. As shown in figure 4B, in the subset of 4 rats tested up to 80 days, the cold hyperalgesia persisted for the entire trial duration.

### Mechanical Withdrawal Testing

**Figure 5:**
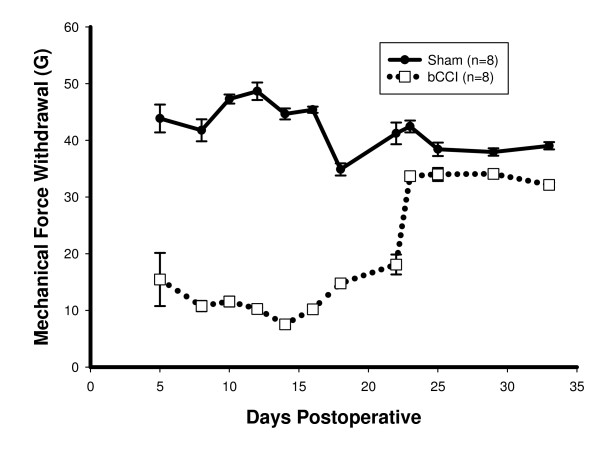
**Hindpaw withdrawal responses to plantar probing with von Frey filament**. Pressure was applied to each hindpaw with electronic von Frey filament. Applied force at the moment of paw withdrawal was determined. Rats were pre-tested to acclimatize to testing protocol. Statistically significant increased reflex withdrawal to mechanical stimuli returned to baseline at day 23 (p < 0.001; F = 801.17; df = 1; two-way ANOVA).

Figure 5 shows the results of probing the plantar surface of the hindpaws with an electronic "von Frey hair" that measured the mechanical force (g) being applied at the time of paw withdrawal. bCCI rats showed significantly lower withdrawal thresholds (p < 0.001; F = 801.17; df = 1; two- way ANOVA) between day 5 and 23 followed by return to control threshold values.

### Immunocytochemistry results

#### CCK-8

**Figure 6:**
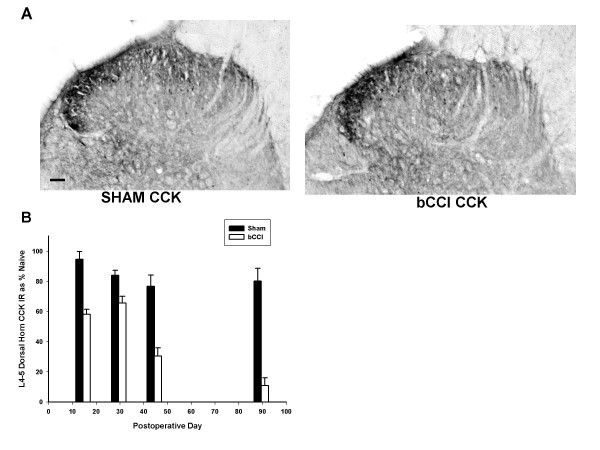
**Superficial dorsal horn immunohistochemical staining for CCK**. (A) Representative photomicrographs of dorsal horn immunoreactivity for cholecystokinin (CCK) in sham operated rats (Sham CCK) versus bCCI rats on post-ligation day 15. Scale bar represents 100 μM. Medial area of the dorsal horn shows greatest decrease in staining in bCCI rats compared to sham-operated controls. (B) Dorsal horn CCK staining densitometry at various postoperative days compared as a percentage of staining in naïve rats. CCK peptide staining decreased at all the time points studied (p < 0.001; F = 68.3; df = 1; Two way ANOVA). CCK -8 showed decrease in immunoreactivity in day 15 (p = 0.001; post hoc Tukey Test) followed by a slight increase at day 30 (p = 0.02; post hoc Tukey test) and then a marked and consistent decrease thereafter to day 90 compared to sham operated rats (p < 0.001; post hoc Tukey test). Data points are group means +/- SEM.

Figure 6 shows that CCK peptide staining decreased at all the time points studied (p < 0.001; F = 68.3; df = 1; two- way ANOVA; Figure 6A and 6B). CCK -8 staining decreased by day 15 (p = 0.001; *post hoc *Tukey Test) followed by a minimal increase at day 30 (p = 0.02; *post hoc *Tukey test) and then a marked and consistent decrease thereafter up to 90 days postoperatively compared to sham operated rats (p < 0.001; *post hoc *Tukey test).

#### Mu Opioid Receptor (MOR)

**Figure 7:**
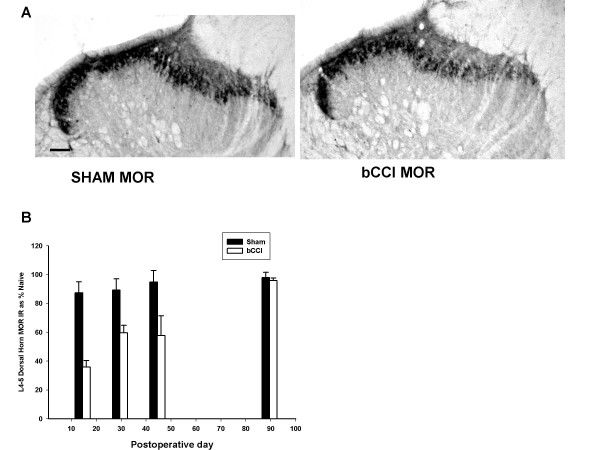
**Effect of bCCI on mu opioid receptor (MOR) staining in superficial dorsal horn**. (A) Representative photomicrographs of dorsal horn immunoreactivity for Morphine Receptor (MOR) in sham operated rats (Sham MOR) versus bCCI rats on post-ligation day 15. Scale bar represents 100 μM. (B) Dorsal horn MOR staining densitometry at various postoperative days after bCCI and sham (control) s surgery as a percentage of staining in naive rats. MOR staining decreased, with maximal decrease at day 15 (p < 0.001; F = 51.38; df = 1; Two way ANOVA) and then gradual increase in day 30 (p = 0.004 compared to sham operated rats post hoc Tukey test) and day 45 (p < 0.001; bCCI compared to sham operated rats post hoc Tukey test). MOR was similar to sham operated rats at day 90. Data points are group means +/- SEM.

Figure 7 shows that Mu opioid receptor staining decreased, with the maximal decrease at day 15 (p < 0.001; F = 51.38; df = 1; two -way ANOVA). Subsequently, MOR staining in bCCI rats gradually increased but remained less than sham-operated rats at day 30 (p = 0.004 compared to sham operated rats; *post hoc *Tukey test) and at day 45 (p < 0.001; bCCI compared to sham operated rats; *post hoc *Tukey test). By day 90, MOR staining was not significantly different in bCCI and sham-operated rats (Figure 7A and 7B)

#### NPY

**Figure 8:**
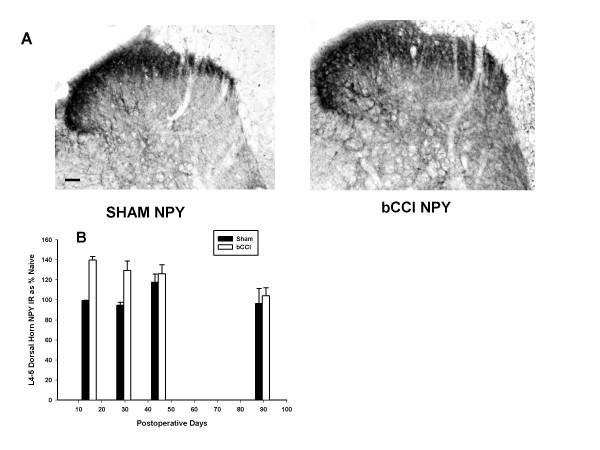
**Effect of bCCI on NPY peptide staining in superficial dorsal horn**. (A) Representative photomicrographs of dorsal horn immunoreactivity for Neuropeptide Y (NPY) in sham operated rats (Sham NPY) versus bCCI rats on post-ligation day 15. (B) Dorsal horn staining densitometry for NPY at various times post-ligation compared to staining in naive rats. NPY peptide showed a consistent statistically significant increase in immunoreactivity (p = 0.003; F = 11.78; df = 1; two-way ANOVA) with maximal increase on post operative day 15. Results were statistically significant for day 15 (p = 0.007; *post hoc *Tukey test) and day 30 (p = 0.023; *post hoc *Tukey test). Data points are group means +/- SEM.

Figure 8 shows that NPY peptide staining initially increased (p = 0.003; F = 11.78; df = 1; two-way ANOVA) with maximal increase on post operative day 15 (Figure 8A and 8B). The difference between bCCI and sham-operated rats was statistically significant for day 15 (p = 0.007; *post hoc *Tukey test) and day 30 (p = 0.023; *post hoc *Tukey test).

#### NK-1 Receptor

**Figure 9:**
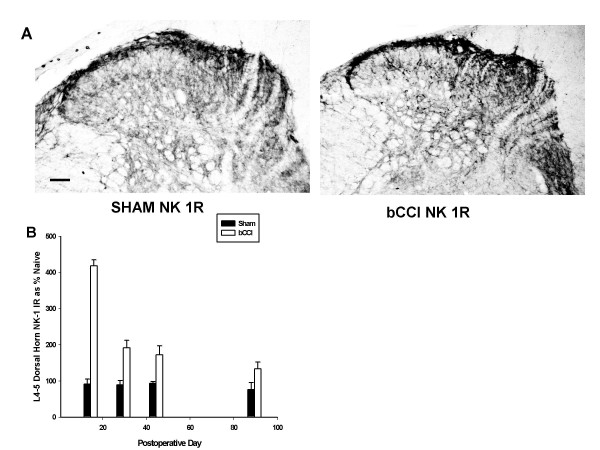
**Effect of bCCI on Neurokinin-1 Receptor (NK-1R) staining in superficial dorsal horn**. (A) Representative photomicrographs of dorsal horn immunoreactivity for Neurokinin-1 Receptor (NK-1R) in sham operated rats (Sham NK 1R) versus bCCI rats on post-ligation day 15. Scale bar represents 100 μM. (B) Dorsal horn staining densitometry for NK-1 R at various postoperative days expressed as a percentage of naive rats. NK-1 receptor showed a consistent statistically significant increase in immunoreactivity, (p < 0.001; F = 145.5; df = 1; two-way ANOVA) with maximal increase on day 15 (increase of greater than 400% of naïve control). Results were statistically significant for all time points studied. [Day 15 and 30 (p < 0.001; *post hoc *Tukey test); 45 day (p = 0.003; *post hoc *Tukey test) and day 90 (p = 0.027; *post hoc *Tukey test)]. Data points are group means +/- SEM.

Figure 9 shows that NK-1 receptor (NK-1R) staining increased (p < 0.001; F = 145.5; df = 1; two-way ANOVA) with maximal increase on day 15 (increase of greater than 400% of control) followed by gradual return toward control levels. Differences between bCCI and sham-operated controls were statistically significant for all time points studied. [Day 15 and 30 (p < 0.001; *post hoc *Tukey test); 45 day (p = 0.003) and day 90 (p = 0.027)].

#### Correlation between behaviour and anatomy

Table 1 shows correlation analyses between cold behaviours for each individual rat and corresponding individual rat densitometry findings that were carried out for day 45 (after mechanical hypersensitivity had resolved). Strong correlations were observed between staining for CCK or for NK-1R and individual rat responses on all cold tests (acetone, cold plate events, duration and latency and thermal preference (0°C vs. 45°C) cold time and crossovers). MOR and NPY did not demonstrate significant correlations with any of the cold behaviours at 45 days postoperatively.

**Table 1:** Correlation/regression analysis for anatomic marker densitometry values vs. cold behavior at postoperative day 45.

Receptor/Peptide	Acetone	L/G events	L/G duration	L/G latency	Place preference crossover	Place preference cold time
CCK	R = 0.84; p = 0.0086	R = 0.94; p = 0.0005	R = 0.89; p = 0.002	R = 0.93, p = 0.006	R = 0.79; p = 0.01	R = 0.86; p = 0.005
MOR	R = 0.46; P = 0.24	R = 0.6; P = 0.11	R = 0.68; P = 0.06	R = 0.67; P = 0.06	R = 0.6; P = 0.11	R = 0.56; P = 0.14
NPY	R = 0.21; p = 0.6	R = 0.28; p = 0.48	R = 0.22; p = 0.59	R = 0.16; p = 0.7	R = 0.5; p = 0.2	R = 0.43; p = 0.28
NK-1R	R = 0.71; p = 0.04	R = 0.83; p = 0.009	R = 0.86; p = 0.005	R = 0.85; p = 0.006	R = 0.79; p = 0.01	R = 0.74; p = 0.03

Highly significant and strong correlations were found between CCK and or NK-1 R and all 6 cold behaviour tests responses (acetone, thermal cold plate 0.3°C lick/guard events; duration and latency and thermal place preference (0 vs. 45°C) crossovers and cold time. MOR and NPY did not demonstrate statistically significant correlations. L/G = lick/guard

## Discussion

The key findings in the present study are: 1 - Sprague Dawley rats subjected to bCCI show prolonged increase in sensitivity to cold stimuli; 2 - enhanced sensitivity to mechanical stimulation is transient in the same bCCI rats that showed persistently enhanced cold sensitivity long after recovery of mechanical sensitivity; and 3 - superficial dorsal horn staining for MOR and NPY peptide are not significantly correlated to behavioural responses to cold stimuli, while CCK-8 and NK-1R were significantly correlated at 45 days. The results of the present study provide evidence that responses to cold stimuli, both reflex and operant responses, are more robust and long lasting than reflex withdrawal responses to mechanical probing in the bCCI model and further suggest that the usefulness of the CCI as a model of neuropathically altered pain sensitivity may be enhanced by use of bilateral lesions coupled with analysis of responses to cold stimuli, particularly operant responses. In the present study, bCCI did not produce evidence of spontaneous pain behaviours, either in home cages or during evoked pain testing.

Our study has several important differences from the previous study by Vierck et al [6]. In the bCCI model, mechanical hyperalgesia returns to baseline by day 25 following surgery. Because we used Sprague Dawley rats, we focused cold plate testing on 0, 0.3, 5 and 10°C compared to the 0.3, 10, 43, 44, 47°C used by Vierck with Long Evans rats. Thermal place preference testing was performed at 0.3° vs. 45°C (vs. 10 and 45°C used by Vierck) which was necessary because we found that the Sprague Dawley rats did not respond to a plate temperature of 10°C. This is likely due to the strain difference (Sprague Dawley vs. Long Evans). Responses to acetone application, a test not evaluated by the Vierck et al group, were particularly robust and present throughout the extended trial period of 45 days (up to 90 days in a subgroup of 4 rats) further supporting the conclusion that both reflex and operant responses to cold are enhanced in bCCI rats. An attractive explanation for this effect on both reflex and operant responses is the very reasonable possibility that enhanced cold responses result from changes in the peripheral nerve and/or superficial dorsal horn, common structures shared by the neural circuitry for both types of responses. Consequently, we chose initially to examine correlations between changes in cold behaviour responses and changes at in superficial dorsal horn anatomy over time.

### Comparison of unilateral CCI to bilateral CCI

In the present study, bCCI rats responded differently than the typical unilateral CCI. For example, there are numerous reports of reduced withdrawal latencies of a CCI limb relative to uninjured contralateral limb from a variety of stimuli including heat, cold and mechanical stimulation [1,32]. Vierck et al [6] showed a shift to heat preference (relative cold aversion) for a minimum of 100 days. The present study corroborates these findings for at least 90 days after ligation suggesting permanently changed responsiveness to cold. In contrast, uCCI studies report latency and thresholds of limb withdrawal from heat, cold and mechanical stimuli are reduced for only a limited time after ligation surgery, peaking within two weeks and lasting less than 40 days [32-36]. In unilateral CCI rats, abnormal postures of the ipsilateral limb also have been observed for a similar duration [33,35,37], suggesting that the time course of withdrawal reflex enhancement in rats with unilateral CCI may be dictated by asymmetric influences on postural control.

Because the CCI procedure can produce damage to axons of motoneurons and axons of afferents that contribute to reflex maintenance of extensor tone during weight bearing [36,38-40], there can be a subtle, unilateral motor deficit coupled with enhancement of flexor tone in the nerve injured limb. Therefore, weight bearing by the nerve injured limb is impaired and withdrawal of the CCI limb from stimulation is favored. Reciprocal flexion and extension of opposing limbs can occur in response to natural stimulation [41]. Furthermore, tonically asymmetric postures can be produced by repetitive cutaneous stimulation [42]. Thus, in unilateral CCI rats, extensor tone of the contralateral, normal limb can be enhanced, interfering with withdrawal of the normal limb and reducing weight transfer to the injured side necessary for lifting responses of the intact hindpaw. These postural adaptations occur within the framework of reciprocal segmental innervation and modulation as a result of unilateral injury producing asymmetrically altered sensory input.

### Comparison of reflex withdrawal vs. operant responses

Clinically relevant study of enhancement of **pain **sensations (allodynia and hyperalgesia) requires behavioural methods that entail cerebral processing of nociceptive input [43]. Operant responses to noxious or aversive stimuli provide the opportunity to observe cerebrally mediated behaviour. For example, long term enhancement of escape responses to cold stimulation after bCCI is reminiscent of the prolonged cold hypersensitivity (hyperalgesia, allodynia) seen in patients after nerve injury [44]. On the other hand, lifting/guarding responses can be elicited in decerebrate but not spinal animals [45]. Whereas, limb withdrawal (flexion) reflexes can be seen in spinal animals [46], and although these responses can be modulated by supraspinal processing, cerebral cortical participation is not required as it is for operant nocifensive responses. Thus, sometimes similar, but often different, results can come from studying reflex vs. operant responses to nociceptive stimuli. For example, operant escape responses and reflex responses differ substantially after experimental manipulations such as systemic morphine [47], spinal cord injury [48], acute stress [49] and destruction of NK-1 receptor-expressing spinal dorsal horn neurons [50]. Interestingly, Jabakhanji et al [51] showed that operant responses can differ between inflammatory and neuropathic pain models. In their thermal challenge test, at both 42° and 35°C, Sprague Dawley rats in the unilateral SNL (spinal nerve ligation) model showed greater preference for a cooler chamber in comparison to the carrageenan (inflammatory) group, a possible difference between unilateral SNL and bCCI models.

### Time course of Behavioural Changes and correlation to Anatomy

We found that behavioural changes do not necessarily mirror some anatomical changes in the dorsal horn. Of the four anatomical markers studied, analyses of individual rat behavioural responses and anatomic measurements at 45 days post ligation revealed statistically significant (p < 0.05) correlations (for CCK, R^2 ^= 0.62-0.88 with p = 0.0005-0.01, and for NK-1R, R^2 ^= 0.50-0.74 with p = 0.006-0.04) between behavioural responses to cold stimuli and staining for CCK and NK-1R, albeit in opposite directions. This information is unique in the sense that the data was obtained from a behaviourally interesting, relatively new model of neuropathic pain (the bCCI model) and correlations obtained at the long time frame of 45 days may be more representative of chronic neuropathic pain in humans, which tends to be persistent and long lasting. Recent work by Polgar et al [52] have demonstrated that significant loss of GABAergic or glycinergic neurons is not necessary for development of thermal hyperalgesia in the unilateral CCI model of neuropathic pain 2 weeks after CCI. The same group also suggested [53] that there was no significant loss of inhibitory interneurons at two weeks from ipsilateral dorsal horn in rats that had undergone unilateral CCI. Thus, the information presented at 45 days correlating cold behavior to the two markers (CCK and NK-1R) may represent inherent plasticity of neuronal responses rather than "receptor and/or cell death".

### Comparion of anatomic changes to other published work on different rat models of neuropathic pain

#### CCK

Zhang and colleagues [54] have shown in dorsal root ganglia that mRNA for both prepro-CCK and its receptor (CCK-2, or CCK-B) are increased by axotomy. However, others [20,55,56] have reported that peripheral axotomy induces a moderate decrease in cholecystokinin-like immunoreactivity in the ipsilateral dorsal horn of the spinal cord. Our results clearly show that cholecystokinin CCK-8 staining is decreased significantly in the dorsal horn of bCCI rats compared to controls for at least 90 days, and this decrease in dorsal horn CCK staining correlates significantly with behavioural responses to cold. Although the physiologic significance and mechanism of these post-injury decreases in dorsal horn CCK are currently obscure, previous reports of the anti-nociceptive effects of CCK antagonists [57-61]suggest that dorsal horn CCK may well be involved in chronic neuropathic pain.

#### MOR

It is a well known clinical observation that opioids often do not provide reliable pain relief in neuropathic pain, but results of the present study do not suggest this insensitivity is related to change in abundance of MOR in the dorsal horn. Porreca and colleagues [20] came to a similar conclusion in a detailed study of MOR in the SNL model. Besse [62] (up to 15 weeks after CCI) and Goff (up to 28 days after CCI) [34] noted up-regulation of MOR in the ipsilateral dorsal horn. The present study shows that MOR staining decreases acutely in the bCCI model followed by a gradual rise back to pre-operative levels by 90 days, even though cold hyperalgesia/allodynia persisted without sign of recovery up to 90 days after CCI in Sprague Dawley rats. Similarly, Vierck noted that enhanced cold responses persisted for at least 100 days in Long Evans rats. In summary, the present study with bCCI and previous reports with uCCI call into question any causal or functional relationship between abundance of MOR in the superficial dorsal horn increased cold sensitivity in bCCI rats.

#### NPY

NPY also is thought to play a role in neuropathic pain. Smith et al [63] studied electrophysiologic and behavioral effects of NPY in spinal cord and dorsal root ganglia and reported that intrathecal NPY reduced nocifensive reflex responses in models of acute inflammatory and neuropathic pain. Ma and Bisby [64] studied dorsal horn and dorsal root ganglion NPY after 3 models of unilateral sciatic nerve injury (partial transaction, complete nerve transaction and chronic constriction injury of the sciatic nerve). In all three models, NPY was dramatically increased in laminae I and II. The present study also demonstrates an increase in NPY levels staining in laminae I-II of the dorsal horn following bCCI for up to 30 days after bCCI similar to the work by Munglaini et al [65] in which uCCI showed increased NPY but in laminae III-IV which persisted for up to 120 days, long after mechanical sensitivity had returned to normal. In the present study, NPY changes do not correlate with persistently enhanced behavioural responses in individual rats at day forty-five thus weakening any inference about the any relationship between bCCI-induced changes in dorsal horn NPY abundance and changes in cold nociception. We agree with Manglaini et al [65] that NPY levels do not reliably relate to alterations in behavioural responses after CCI. Combined with the present results, it seems likely that NPY changes are related more to changes in mechanical responses, rather than cold nociception, and dorsal horn levels of NPY, not surprisingly, do not predict effects of exogenous NPY drugs as reported by Intondi et al [66].

#### NK-1R

Doyle and Hunt [67] found that dorsal horn neurokinin-1 receptor (NK-1R) cells encode for the intensity of noxious cooling of the skin suggesting a rationale for studying superficial dorsal horn NK-1R staining in bCCI rats. We found that NK-1R increased significantly for all time points studied. Additionally, the increase in NK-1R correlated well with changes in behavior at 45 days. Thus, NK-1R-expressing dorsal horn neurons may also play a role in chronic neuropathic pain syndromes. Certainly, selective destruction of superficial dorsal horn neurons expressing NK-1R is powerfully anti-nociceptive as suggested by the reported anti-nociceptive effects of intrathecal substance P-saporin [68,69] which irreversibly destroys these neurons and reduces nocifensive reflex responses to mechanical stimulation in several models of neuropathic and inflammatory pain [70].

#### Phases of the CCI Model

Some investigators have suggested that Wallerian degeneration and macrophage infiltration as a cause of early neuropathic pain. Certainly, the thermal hyperalgesia following uCCI in normal animals peaks at the time of maximum affected macrophage infiltration of the nerve [71], and there is a clear correlation between the number of macrophages in the nerve and the withdrawal threshold to mechanical stimulation [72]. Some evidence implicates inflammatory mediators in early stages of the uCCI model (first month) including TNF-alpha and cytokines [73]. We propose that this early neuroinflammatory response to bCCI may play a role in the transient enhancement of mechanical responses and is replaced by a late phase after inflammation has resolved, that is characterized by long lasting cold hyperalgesia and allodynia.

## Conclusions

Compared to human subjects with neuropathic pain, responses to cold in rats with bCCI may be a useful model of neuropathic pain. In particular, we propose that the bCCI model consists of two phases: an early phase characterized by increased mechanical and cold sensitivity, and a later phase (beyond 30 days) characterized by long lasting cold hyperalgesia and allodynia. Persistently elevated NK-1R and decreased CCK expression in the superficial dorsal horn correlate with the abnormal cold sensitivity. The decrease in CCK raises the possibility that manipulation of dorsal horn CCK receptor-expressing neurons may be a useful approach to neuropathic pain.

## Methods

Experiments were performed with female Sprague Dawley rats. The rats were housed in pairs in an AAALAC and USDA approved facility in shoebox plastic cages with soft, loose bedding. Food and water were supplied *ad libitum*, and a 12-hour light/dark cycle was maintained. Animal care and use conformed to National Institutes of Health guidelines for care and use of experimental animals. Experimental protocols were approved by the Vanderbilt University Institutional Animal Care and Utilization committee. Behavior testing and anatomic analyses were conducted in blinded fashion such that personnel responsible for collecting data did not know which treatment group a rat or a set of slides came from.

### Bilateral Sciatic Nerve Ligation and Sham Surgery

bCCI of the sciatic nerves was performed under aseptic conditions. The rats were anesthetized with an intraperitoneal mixture of ketamine (80 mg/kg) and xylazine (3 mg/kg) and acepromazine (0.75 mg/kg). The sciatic nerve on each side was exposed through a mid-thigh incision and separation of the heads of the biceps femoris muscle. Each sciatic nerve was identified above the trifurcation and freed from surrounding loose connective tissue. Three snug ligatures of 4-0 chromic gut were placed around the nerve. The sutures were placed with just enough pressure to produce mild blanching of the epineurium visible under the operating scope. Sham surgery was identical except that no ligatures were placed on the sciatic nerves. A single individual (SD) performed all the sham and bilateral sciatic nerve ligations.

### Cold plate testing

Rats were placed in a 6" × 9" × 12" high plexiglass enclosure. The floor was a custom-made aluminum plate with internal channels for circulation of heated or cooled water, supplied by a thermostatically controlled circulator (model RET-111; Neslab, Newington, NH). Plate surface temperatures of 0.3°, 5°, 10° or 45°C were maintained and continuously monitored by using a contact temperature probe connected to a model 4900 thermometer (Yellow Springs Instruments, Yellow Springs, Ohio). A moderate level of illumination (15.5 foot candles) was present during reflex testing. During reflex testing, one investigator (KC) observed the animals for ten minutes and recorded the onset and duration of each hind paw L/G responses by keystroke entries into custom designed computer program.

### Thermal Preference testing (TPT)

For thermal preference testing (TPT), two 6" × 9" plexiglass compartments separated by a partition with a 2.5- by 2.5-inch opening permitting access to either compartment was used to make a simple shuttle box. The floor of one compartment was maintained at 45°C and the temperature of the other floor was maintained at 0.3°C. Floor temperatures were randomly switched from side to side each day, resulting in being placed on either the hot or the cold side in random fashion to minimize initial response bias. Illumination of each compartment was equal and low (about 1 foot candle). For each 10-minute trial, rats were randomly placed at the beginning of each trial on either the cold (0.3°C) or the warm (45°C) side to minimize response bias and were monitored for time spent on the cold side and crossovers in either direction from one side to the other.

### Acetone Testing

A drop (0.1 ml) of acetone was gently applied to each hindpaw through a polyethylene (PE) 10 plastic tubing connected to a 1 ml syringe. A brisk foot withdrawal response after the spread of acetone over the planter surface of the hind paw was considered a sign of cold hyperalgesia. The test was repeated 5 times for each hindpaw, alternating hindpaws for a total of 10 trials per day, with interval of approximately 2 minutes between each test. Results were graded as percentage of applications that evoked a response of paw withdrawal. Increased percentage of applications eliciting a withdrawal response compared to control was interpreted as development of increased cold sensitivity.

### Mechanical hyperalgesia testing

Mechanical hyperalgesia was tested using an electronic von Frey filament (IITC Life Sciences, Woodland Hills, CA). Rats were acclimated to mesh bottom cages for 5-15 minutes. Testing consisted of applying pressure to the plantar surface of each hind paw from below with the electronic von Frey filament through the mesh floor, alternating hindpaws for a total of 10 trials per day (five per hindpaw). The force applied at the time of paw withdrawal was recorded. Rats were pre-tested before ligation surgery to acclimate them to the testing protocol. Pretesting was carried out for at least three consecutive days before bilateral chronic constriction injury surgery.

### Immunocytochemistry Protocol

#### Tissue preparation

Rats were deeply anesthetized with sodium pentobarbital and perfused transcardiacally with 200-300 ml of cold normal saline containing 5 mM sodium phosphate, pH 7.5, 1 g/l sodium nitrite (vasodilator) and 1000 units/l sodium heparin (anticoagulant) followed by 4% formaldehyde prepared from paraformaldehyde in 100 mM sodium phosphate, pH 7.5. Spinal cords were postfixed for at least 1 h and stored in fixative at 4°C. The day prior to sectioning, lumbar enlargement spinal cord blocks were equilibrated overnight in 30% sucrose in 5 mM sodium phosphate, pH 7.5. 40 μM transverse sections of the lumbar enlargement are cut on a freezing stage of a sliding microtome (American Optical) and collected in PBS in groups of six sections/well of 24- well tissue culture plates. For storage at -70°C, sections are equilibrated with "antifreeze" consisting of glycerol-ethylene glycol-phosphate buffer.

#### Immunohistochemical procedures

All control and bCCI sections were stained in parallel with control sections using the same reagents and, solutions and dilutions. 7 to 10 randomly selected sections from spinal segments L4 and L5 were removed from antifreeze at room temperature and washed in Tris-buffered saline followed by incubation for 1 h in 5% normal serum at room temperature. Then the free-floating sections were transferred to primary antibody solution and incubated overnight at 4°C.

The next day sections were washed and processed for peroxidase immunohistochemistry using the standard biotin-avidin technique (ABC elite kit, Vector laboratories, Burlingame, CA, USA) using diaminobenzidine/nickel as chromogen. Primary rabbit anti- MOR antibody and rabbit anti-NK-1 R antibody was obtained from Chemicon International (Temecula, CA, USA), rabbit anti-NPY antibody from Peninsula International (San Carlos, CA, USA) and rabbit anti- CCK peptide from Sigma Chemicals (St. Louis, MO, USA). Reacted sections were washed and mounted on gelatin coated slides, dehydrated, cleared and examined using a Leitz Orthoplan research microscope with digital camera.

#### CCK-8, MOR, NPY and NK-1R measurements

As previously reported [50,74-76]; we used computer assisted quantitative densitometry to evaluate CCK-8, MOR, NPY and NK-1R staining in the superficial dorsal horn. Using randomized coded sections to blind the operator to experimental condition, user-defined areas of interest encompassing the entire medio-lateral extent of lamina I and II were digitally captured. Both right and left dorsal horns from 8 to 10 sections of lumbar segments L 4 and L5 from each spinal cord were photographed. After correction for any differences in background intensity, the darkest pixels (intensity 0-100 out of a range of 0-250 gray levels) were chosen as consistently representing specific staining when compared with the distribution of computer selected stained pixels by visual inspection of the peroxidase stained sections. Mean pixel counts for each rat were computed from the 8 to 10 L4 and L5 sections measured from each rat. All measurements were performed on raw digital image, no transformation or manipulations were applied to the original image from which all measurements were taken.

### Data Analysis procedures

#### Statistical procedures

Raw behavior data (number of nocifensive responses, duration of nocifensive or TPT response time etc) were analysed using Student's t-test, ANOVA (one- and two-way with repeated measures) and in some cases non-parametric rank-based tests (rank sum, signed rank and ANOVA on ranks) were used when data deviated significantly from the normal distribution as determined by the Kolmogorov-Smirnov criteria (for p ≤ 0.05).

Raw anatomic data (cell counts, stained pixel counts) were compared primarily using two-way repeated measures ANOVA techniques with minimum significance level of p ≤ 0.05 to reject the null hypothesis. The Tukey test was used for pair-wise comparisons of group means within the ANOVA analysis. Standard Pearson product-moment or non-parametric Spearman rank order correlation (if data were not normally distributed) coefficients were calculated for analysis of interrelationships between variables. Raw densitometry values of the two groups (sham operated and bCCI) were compared to naïve controls and data expressed as percentage of naïve controls.

Statistical calculations and comparisons used Sigma Stat software (SPSS Inc., Chicago, IL).

## Competing interests

RGW is chief scientific advisor to Advanced Targeting Systems, San Diego, CA, supplier of CCK-saporin.

## Authors' contributions

SD conducted bCCI and sham ligation surgery, participated in development of standardized protocols for behavioral anatomical and behavioral models, performed statistical analysis and drafted the manuscript. KC performed all the experiments, performed statistical analysis. RHK assisted in behavioral and anatomical experiments. RGW participated in design of the study, statistical design and interpretation and helped co-write to draft the manuscript. All authors read and approved the final manuscript.
